# Supplementary material for: Exploring mechanisms that affect coral cooperation: symbiont transmission mode, cell density and community composition
Source: PeerJ. 2018 Dec 3;6:e6047. doi: 10.7717/peerj.6047 (PMC6282938; doi:10.7717/peerj.6047)
Supplement: Supplemental Information 1 — Additional supplementary material as referenced in main manuscript text. [file peerj-06-6047-s001.docx]

**Supplementary Material: Exploring mechanisms that affect coral cooperation: symbiont transmission mode, cell density and community composition**

Carly D. Kenkel and Line K. Bay

Table S1. Raw read counts and reads lost during filtering and clustering with DADA2.

| **Sample** | **Raw** | **Input_DADA2** | **filtered** | **denoised** | **merged** | **tabled** | **nonchim** |
| --- | --- | --- | --- | --- | --- | --- | --- |
| AmilAll | 35820 | 35061 | 31194 | 31194 | 28315 | 28315 | 24013 |
| Gach1 | 74818 | 74054 | 67294 | 67294 | 66374 | 66301 | 62896 |
| Gach10 | 72354 | 71449 | 60322 | 60322 | 59915 | 59451 | 50573 |
| Gach2 | 74662 | 74012 | 63885 | 63885 | 62752 | 62250 | 50278 |
| Gach3 | 35176 | 34552 | 25626 | 25626 | 25323 | 25140 | 22602 |
| Gach4 | 93883 | 93234 | 80667 | 80667 | 79625 | 79064 | 63253 |
| Gach5 | 30755 | 30541 | 26595 | 26595 | 26375 | 26167 | 22976 |
| Gach6 | 40510 | 39898 | 34705 | 34705 | 34374 | 34019 | 31309 |
| Gach7 | 4472 | 4332 | 2643 | 2643 | 2611 | 2598 | 2528 |
| Gach8 | 33640 | 33267 | 29026 | 29026 | 28780 | 28443 | 26381 |
| Gach9 | 40512 | 40026 | 34308 | 34308 | 34026 | 33542 | 31669 |
| GachAll | 25286 | 24821 | 21705 | 21705 | 21399 | 21246 | 17958 |
| Gast1 | 1061 | 942 | 582 | 582 | 581 | 581 | 571 |
| Gast10 | 33882 | 33390 | 30573 | 30573 | 30231 | 30212 | 29162 |
| Gast2 | 21197 | 20863 | 18934 | 18934 | 18696 | 18696 | 17876 |
| Gast3 | 20029 | 19875 | 18266 | 18266 | 18003 | 18003 | 17431 |
| Gast4 | 16471 | 16248 | 13906 | 13906 | 13741 | 13558 | 12598 |
| Gast5 | 11943 | 11730 | 10196 | 10196 | 10172 | 10172 | 9182 |
| Gast6 | 45291 | 44813 | 40014 | 40014 | 39595 | 39574 | 38225 |
| Gast7 | 33652 | 33320 | 29961 | 29961 | 29883 | 29883 | 26478 |
| Gast8 | 23892 | 23539 | 21491 | 21491 | 21312 | 21287 | 20885 |
| Gast9 | 734 | 621 | 374 | 374 | 374 | 374 | 337 |
| GastAll | 31819 | 31304 | 27836 | 27836 | 27425 | 27367 | 23894 |
| GcolAll | 10196 | 9940 | 7972 | 7972 | 7839 | 7839 | 7661 |
| Maqe10 | 77568 | 69196 | 44998 | 44998 | 44705 | 44698 | 42679 |
| Maqe6 | 57788 | 46772 | 31275 | 31275 | 31160 | 31114 | 30225 |
| Maqe6b | 51170 | 27633 | 17137 | 17137 | 17074 | 17047 | 16564 |
| Maqe7 | 29361 | 23378 | 16091 | 16091 | 15970 | 15970 | 15338 |
| Maqe7b | 17131 | 16676 | 11653 | 11653 | 11567 | 11567 | 11491 |
| Maqe8 | 72073 | 43668 | 29519 | 29519 | 29346 | 29346 | 27746 |
| Maqe8b | 12976 | 12418 | 8388 | 8388 | 8314 | 8314 | 8214 |
| Maqe9 | 20282 | 19533 | 13318 | 13318 | 13226 | 13226 | 13131 |
| Maqe9b | 59314 | 31986 | 19854 | 19854 | 19715 | 19715 | 19002 |
| MaqeAll | 14445 | 14232 | 11940 | 11940 | 11754 | 11754 | 10322 |
| Plob6 | 16697 | 5454 | 3630 | 3630 | 725 | 584 | 561 |
| Plob6b | 28036 | 3924 | 2461 | 2461 | 1354 | 1294 | 1184 |
| Plob7 | 1469 | 454 | 263 | 263 | 44 | 30 | 3 |
| Plob7b | 17267 | 1798 | 1135 | 1135 | 197 | 109 | 109 |
| Plob8b | 36191 | 19086 | 12600 | 12600 | 11986 | 11940 | 11837 |
| PlobAll | 31619 | 30869 | 27527 | 27527 | 26407 | 26341 | 21754 |

Table S2. Best BLASTn matches to the GeoSymbio ITS2 database (Franklin et al. 2012) for curated amplicon sequence variants (ASVs) detected across samples. Value in % column indicates mean percent abundance across all samples in which the sequence variant, or sub-variants (identified through LULU curation), were identified. Match bit-scores are reported and hits separated by ‘/’ in GeoSymbio column indicate equally high scoring blast matches.

| **ASV** | **%** | **Score** | **GeoSymbio BLAST** |
| --- | --- | --- | --- |
| Sq1 | 31.6 | 523 | C1 |
| Sq2 | 18.8 | 501 | D1 |
| Sq3 | 10.6 | 508 | C35(type2)/C26.b1 |
| Sq4 | 7.0 | 501 | D1a |
| Sq5 | 11.4 | 508 | C35a(type2)/C26a/C26 |
| Sq6 | 6.6 | 512 | C35(type2)/C26.b1 |
| Sq7 | 3.8 | 501 | D6 |
| Sq8 | 4.9 | 523 | C3/Cspc |
| Sq10 | 2.5 | 512 | C35a(type2)/C26a/C26 |
| Sq18 | 1.6 | 520 | C15 |
| Sq23 | 0.8 | 523 | C15 |
| Sq40 | 0.3 | 523 | C15a |

Table S3. Wald test statistics for individual factors and interaction terms for the *Symbiodinium* cell density model. Significant terms are shown in bold.

numDF denDF F-value p-value

----------------- ------ ------ ------------- ----------

(Intercept) 1 270 1.989512e+05 0.0000000

**Species 5 52 9.116880e+00 0.0000028**

**Temp 1 270 1.260908e+01 0.0004528**

**Day 2 270 6.089511e+00 0.0025898**

Species:Temp 5 270 1.104898e+00 0.3580558

Species:Day 10 270 1.452625e+00 0.1573259

**Temp:Day 2 270 1.388694e+01 0.0000018**

Species:Temp:Day 10 270 4.876186e-01 0.8975041

Table S4. Wald test statistics for individual factors and interaction terms for the net photosynthesis model. Significant terms are shown in bold.

numDF denDF F-value p-value

----------------- ------ ------ ------------- ----------

(Intercept) 1 270 1648.4748360 0.0000000

**Species 5 52 40.2840568 0.0000000**

Temp 1 270 3.4308423 0.0650808

**Day 2 270 5.7878681 0.0034574**

Species:Temp 5 270 1.2021088 0.3084131

Species:Day 10 270 0.8998245 0.5338268

**Temp:Day 2 270 36.4032169 0.0000000**

Species:Temp:Day 10 270 1.0755283 0.3486423

Table S5. Wald test statistics for individual factors and interaction terms for the fraction of DPM shared model. Significant terms are shown in bold.

numDF denDF F-value p-value

----------------- ------ ------ ------------- ----------

(Intercept) 1 120 5585.5585773 0.0000000

**Species 5 22 18.7172944 0.0000003**

**Temp 1 120 18.0748728 0.0000423**

**Day 2** 120 **24.4071621 0.0000000**

**Species:Temp 5 120 3.4767095 0.0057086**

Species:Day 10 120 0.6096878 0.8031208

**Temp:Day 2** 120 **14.5121654 0.0000023**

Species:Temp:Day 10 120 0.8893596 0.5453031

Figure S1. Daily measures of effective quantum yield (mean EQY±SEM, note that range of error bars is extremely small, in most cases barely exceeding points) of *Symbiodinium* photosystem II by temperature treatment. Bars show the difference in mean EQY between treatments through time (n=720 per bar).


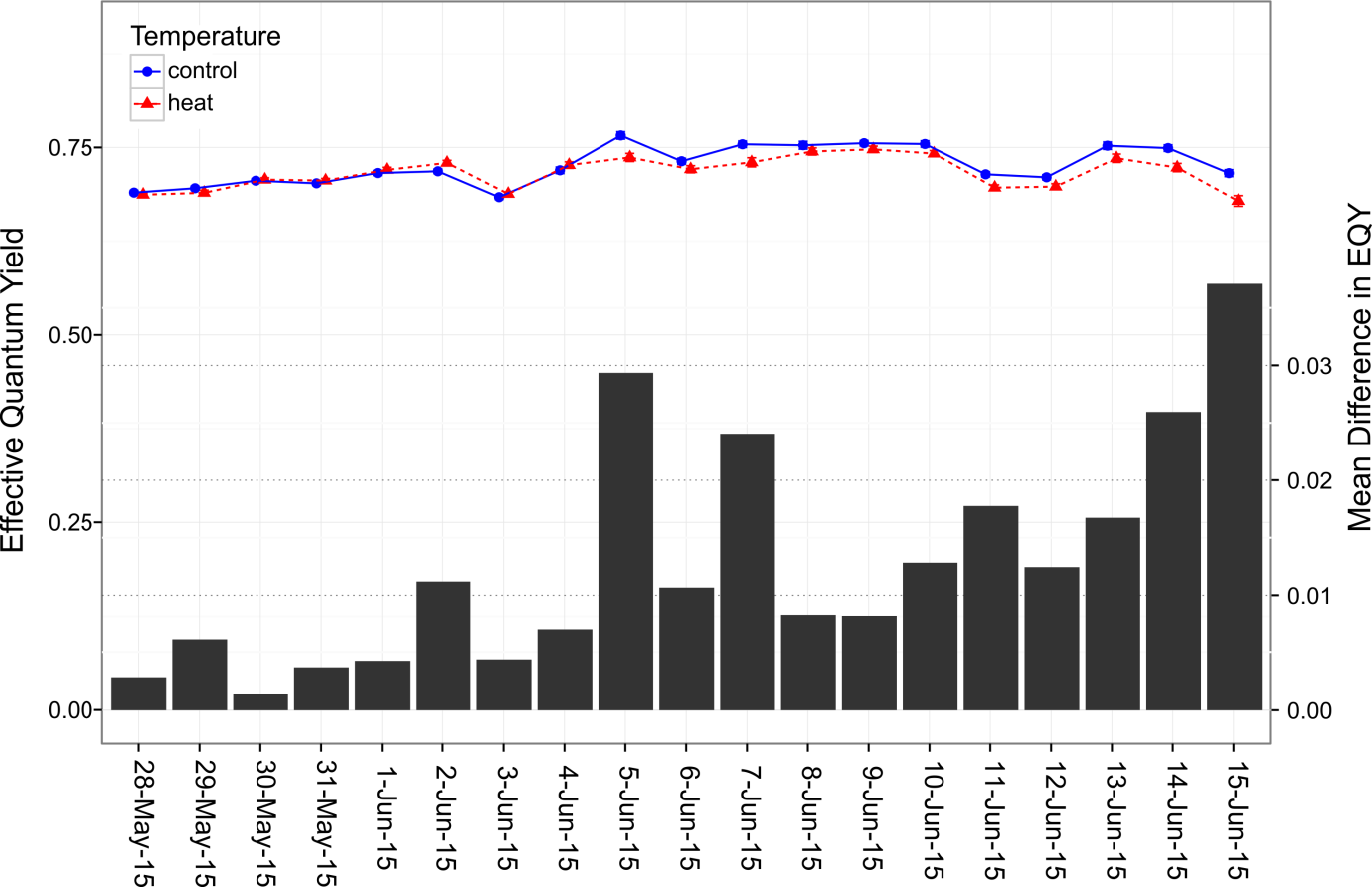


Figure S2. Daily measures of effective quantum yield (EQY±SEM) of *Symbiodinium* photosystem II by temperature treatment and species.

Figure S3. Temperature profile of experimental treatment tanks (±SD) by date. Bars indicate time of sampling.


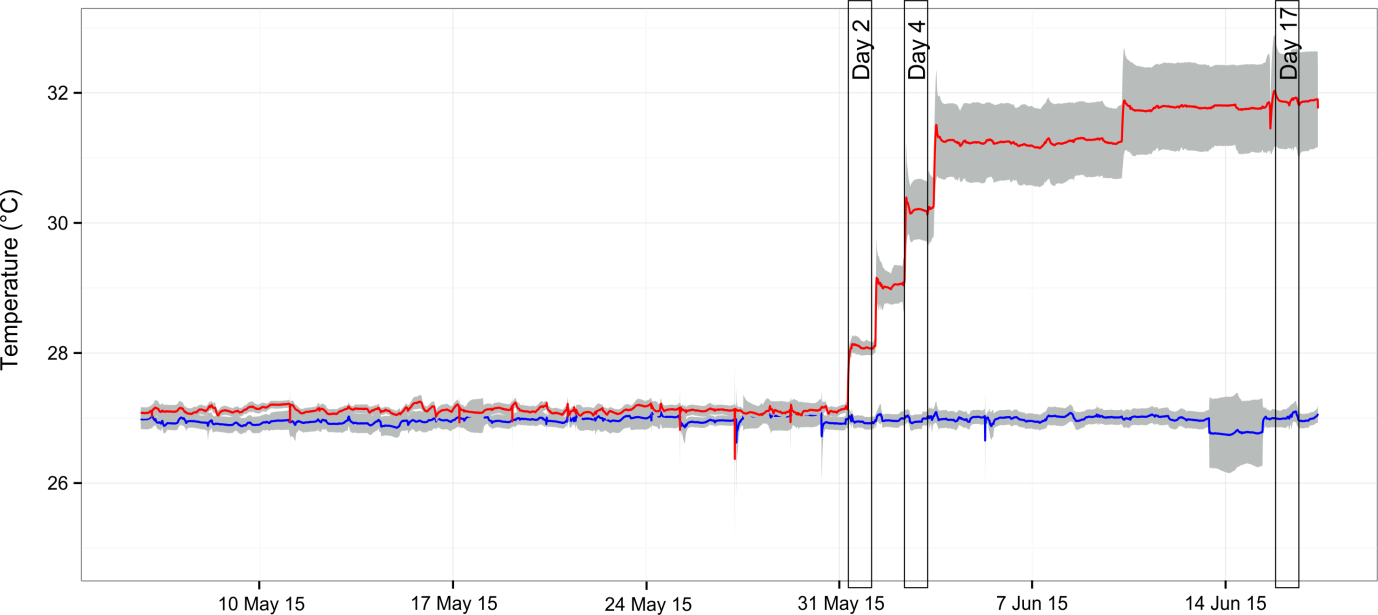


Figure S4. *Symbiodinium* cell density, expressed as cells per cm^2^ of coral surface area of focal coral species (Amil: *Acropora millepora*, Maeq: *Montipora aequituberculata*, Gcol: *Goniopora columna*, Plob: *Porites lobata*, Gast: *Galaxea astreata*, Gacr: *Galaxea acrhelia*) under control (27°C, blue circles) and elevated (31°C, red triangles) temperature following 2, 4 and 17 days of treatment.


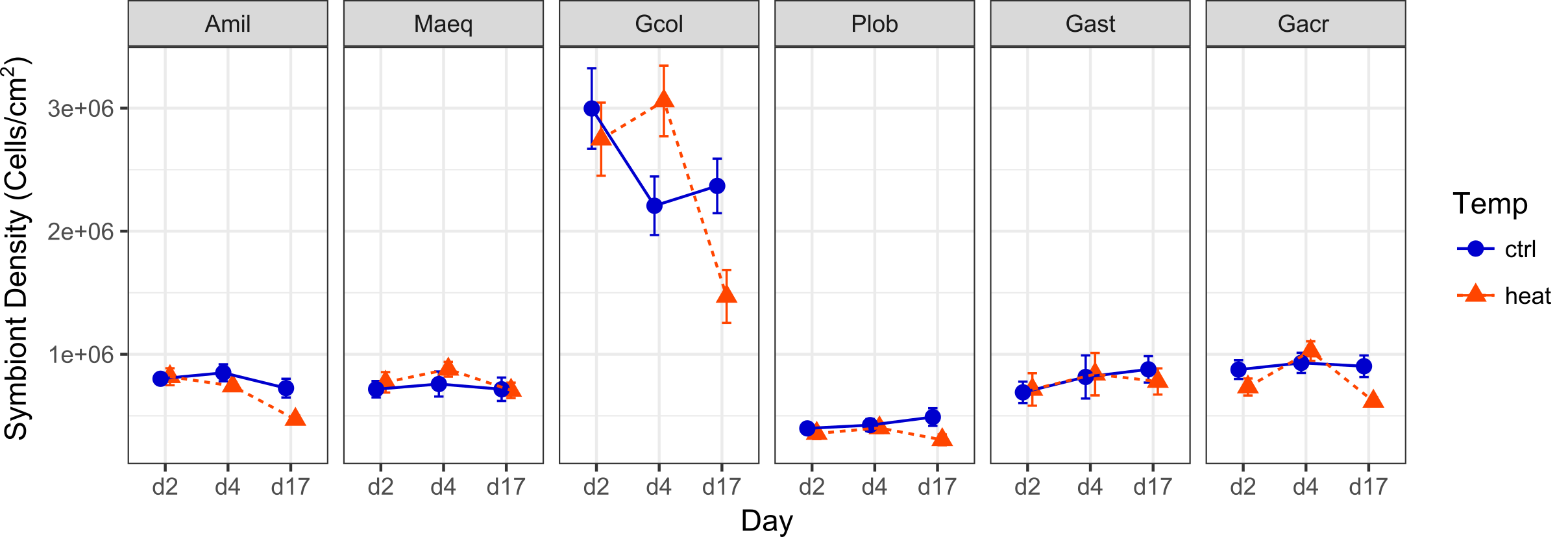


Figure S5. Taq1 digest of *Symbiodinium* LSU rRNA. Samples are grouped by species pairs. Standard shows expected banding pattern for *Symbiodinium* genotypes in clades C and D.


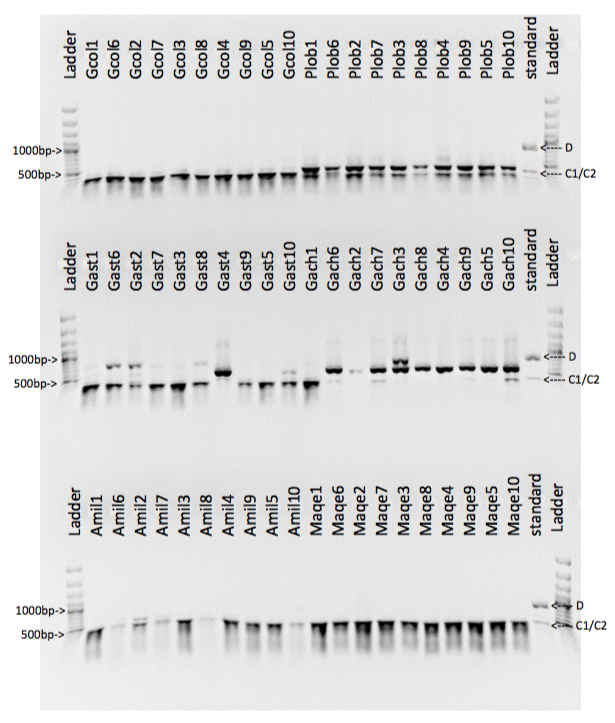


Fig S6. Normalized proportional abundance of sequence variants by samples sequenced at UT Austin’s GSAF (All and individual Gast/Gacr samples) and OSU’s CGRB (individual Maeq and Plob samples). Gacr1 and Gast4 were excluded from subsequent analyses as they appear to be mis-labeled and for analyses comparing differences among species, the ‘All’ samples for Gacr, Gast, Maeq and Plob were replaced by *in silico* averages calculated using the individual sample replicates shown here.


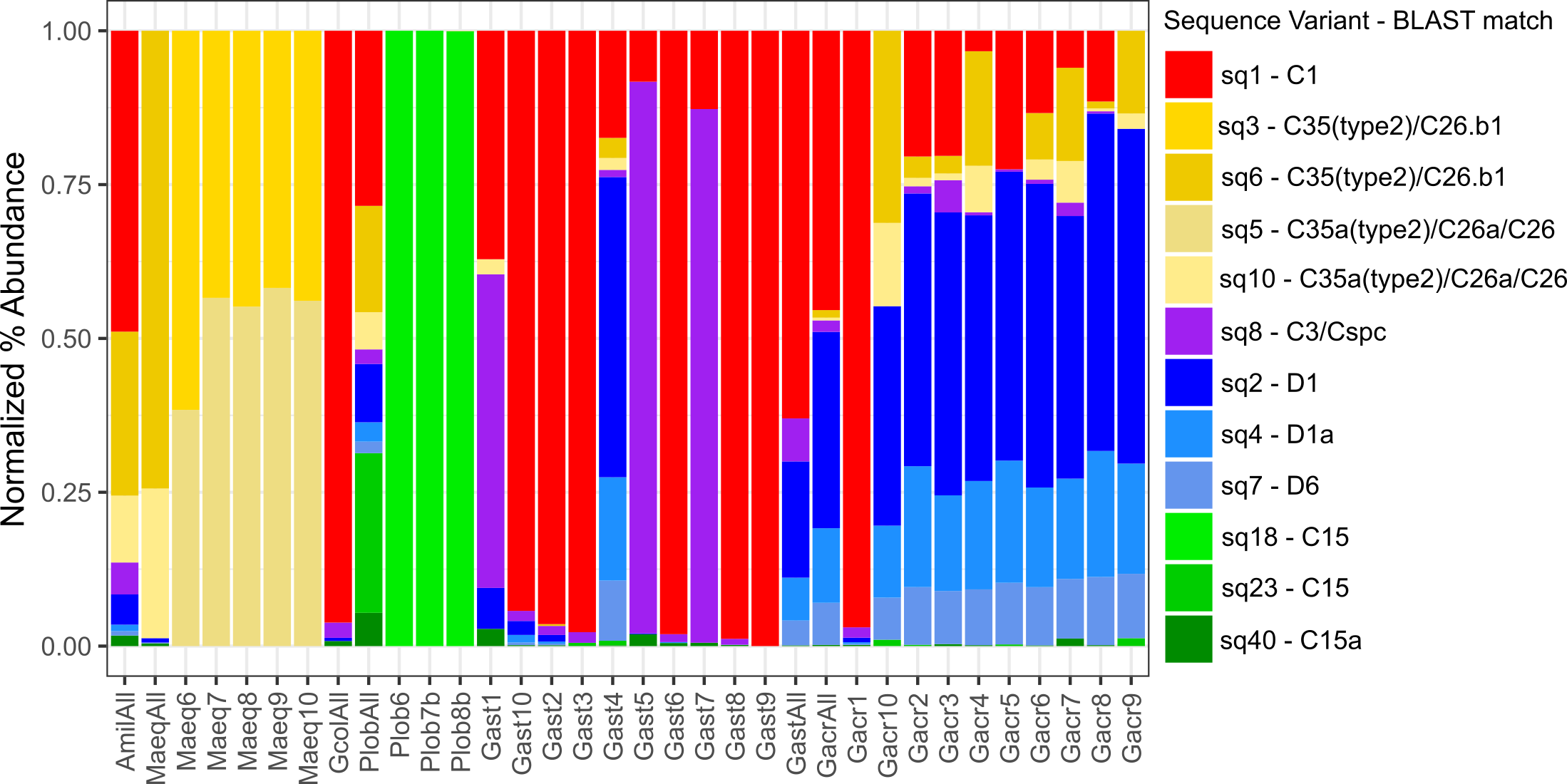


REFERENCES

Franklin, E. C., M. Stat, X. Pochon, H. M. Putnam, and R. D. Gates. 2012. GeoSymbio: a hybrid, cloud-based web application of global geospatial bioinformatics and ecoinformatics for Symbiodinium-host symbioses. Molecular Ecology Resources 12:369-373.

Veal, C. J., M. Carmi, M. Fine, and O. Hoegh-Guldberg. 2010. Increasing the accuracy of surface area estimation using single wax dipping of coral fragments. Coral Reefs 29:893-897.
